# A Clinical Audit to Review the Use of Antipsychotic Medications in Patients With a Diagnosis of Dementia in Community Mental Health Team for Older Adults in West Essex

**DOI:** 10.1192/bjo.2026.11831

**Published:** 2026-06-30

**Authors:** Pavithra Padmanabha Rao, Leonidas Chouliaras

**Affiliations:** 1 Essex Partnership University NHS Foundation Trust, Harlow, United Kingdom; 2 Essex Partnership University NHS Foundation Trust, Epping, United Kingdom

## Abstract

**Aims:**

The use of antipsychotics in behavioural and psychological symptoms of dementia (BPSD) increases the risk of cerebrovascular adverse events and mortality. The aim is to check whether the use of antipsychotic medications for BPSD is in accordance with the NICE guidelines, i.e.,

1. Documentation of specific BPSD.

2. Exclusion of clinical or environmental causes.

3. Use of non-pharmacological interventions prior to commencing antipsychotics.

4. Use of structured assessment such as an ABC chart for assessing BPSD.

5. Antipsychotic review every 6 weeks to consider the need for continuation of antipsychotics.

**Methods:**

The audit was carried out across the care homes, nursing and residential homes under the Older Adult Community Mental Health Services in West Essex (Epping, Uttlesford, and Harlow). The data collection was done between July to September 2025, and the information was retrospectively gathered from SystmOne records between 01/10/2024 and 31/03/2025. The patients with a formal diagnosis of dementia prescribed with antipsychotics were included in the study. They were excluded if prior to the first 6-week review of antipsychotics they were transferred out of area, moved out of a care homes, nursing and residential homes, deceased, or had the antipsychotics discontinued. A specially designed data-collection tool was used to collect the data by the data collectors.

**Results:**

Of the 78 patients assessed, the majority were females and in their 80’s. The most common diagnosis was Dementia in Alzheimer’s disease, mixed type. The documentation of BPSD showed full compliance with the guidelines. There was good compliance (91%) with evidence of investigations undertaken to exclude organic conditions and delirium. However, the documentation regarding excluding environmental causes of BPSD and the use of non-pharmacological methods prior to commencing antipsychotics was unclear and hence could not be audited. The evidence of use of ABC chart for assessing patients with BPSD was found only in 38.5%. Two- thirds of the patients were on Risperidone, followed by Quetiapine (14%) and Aripiprazole (9%). A total of 59 (75.6%) patients received an initial 6-week review and only 43 (55.2%) patients had subsequent reviews at 6-weekly intervals.

**Conclusion:**

The audit revealed that the review of patients with Dementia prescribed with antipsychotics was not found to be concordant with the NICE guidelines. Implementing a standardised antipsychotic review record may help improve documentation and support the setting of a tentative review date, thus reducing non-compliance to the standards and thus ensuring safety and a better quality of life for patients with Dementia.

